# Different states of stemness of glioblastoma stem cells sustain glioblastoma subtypes indicating novel clinical biomarkers and high-efficacy customized therapies

**DOI:** 10.1186/s13046-023-02811-0

**Published:** 2023-09-21

**Authors:** Alberto Visioli, Nadia Trivieri, Gandino Mencarelli, Fabrizio Giani, Massimiliano Copetti, Orazio Palumbo, Riccardo Pracella, Maria Grazia Cariglia, Chiara Barile, Luigi Mischitelli, Amata Amy Soriano, Pietro Palumbo, Federico Legnani, Francesco DiMeco, Leonardo Gorgoglione, Graziano Pesole, Angelo L. Vescovi, Elena Binda

**Affiliations:** 1 StemGen SpA, Milan, Italy; 2 Cancer Stem Cells Unit, Institute for Stem Cell Biology, Regenerative Medicine and Innovative Therapeutics (ISBReMIT), IRCSS Casa Sollievo della Sofferenza, Opera di San Pio da Pietrelcina, San Giovanni Rotondo, FG Italy; 3 Biostatistical Unit, IRCCS Casa Sollievo della Sofferenza, San Giovanni Rotondo, Italy; 4 Medical Genetics Unit, IRCCS Casa Sollievo Della Sofferenza, San Giovanni Rotondo, Italy; 5 Department of Neurosurgery, National Neurologic Institute IRCCS C. Besta, Milan, Italy; 6 Department of Neurosurgery, John Hopkins University, Baltimore, Mariland USA; 7 Department of Oncology and Hemato-oncology, University of Milan, Milan, Italy; 8 Neurosurgery Unit, IRCCS Casa Sollievo della Sofferenza, Milan, Italy; 9 Department of Biosciences, Biotechnology and Environment, University of Bari A. Moro, Bari, Italy; 10 Institute of Biomembranes, Bioenergetics and Molecular Biotechnologies, National Research Council, Bari, Italy; 11 Scientific Directorate, IRCCS Casa Sollievo della Sofferenza, San Giovanni Rotondo, Italy; 12 Hyperstem SA, Lugano, Switzerland

**Keywords:** Glioblastoma, Glioblastoma stem cells (GSCs), Stemness-related therapeutic biomarkers, Anti-GBM patient-tailored strategies

## Abstract

**Background:**

Glioblastoma (GBM) is the most malignant among gliomas with an inevitable lethal outcome. The elucidation of the physiology and regulation of this tumor is mandatory to unravel novel target and effective therapeutics. Emerging concepts show that the minor subset of glioblastoma stem cells (GSCs) accounts for tumorigenicity, representing the true target for innovative therapies in GBM.

**Methods:**

Here, we isolated and established functionally stable and steadily expanding GSCs lines from a large cohort of GBM patients. The molecular, functional and antigenic landscape of GBM tissues and their derivative GSCs was highlited in a side-by-side comprehensive genomic and transcriptomic characterization by ANOVA and Fisher’s exact tests. GSCs’ physio-pathological hallmarks were delineated by comparing over time in vitro and in vivo their expansion, self-renewal and tumorigenic ability with hierarchical linear models for repeated measurements and Kaplan–Meier method. Candidate biomarkers performance in discriminating GBM patients’ classification emerged by classification tree and patients’ survival analysis.

**Results:**

Here, distinct biomarker signatures together with aberrant functional programs were shown to stratify GBM patients as well as their sibling GSCs population into TCGA clusters. Of importance, GSCs cells were demonstrated to fully resemble over time the molecular features of their patient of origin. Furthermore, we pointed out the existence of distinct GSCs subsets within GBM classification, inherently endowed with different self-renewal and tumorigenic potential. Particularly, classical GSCs were identified by more undifferentiated biological hallmarks, enhanced expansion and clonal capacity as compared to the more mature, relatively slow-propagating mesenchymal and proneural cells, likely endowed with a higher potential for infiltration either ex vivo or in vivo. Importantly, the combination of DCX and EGFR markers, selectively enriched among GSCs pools, almost exactly predicted GBM patients’ clusters together with their survival and drug response.

**Conclusions:**

In this study we report that an inherent enrichment of distinct GSCs pools underpin the functional inter-cluster variances displayed by GBM patients. We uncover two selectively represented novel functional biomarkers capable of discriminating GBM patients’ stratification, survival and drug response, setting the stage for the determination of patient-tailored diagnostic and prognostic strategies and, mostly, for the design of appropriate, patient-selective treatment protocols.

**Supplementary Information:**

The online version contains supplementary material available at 10.1186/s13046-023-02811-0.

## Background

Glioblastoma (GBM), which is classified as Isocitrate dehydrogenase (IDH1) wild-type (IDH1wt) according to 2021 WHO [1], is the most common and aggressive form among primary malignant brain tumors. Despite aggressive therapies and advances in genomic and molecular classifications, the five-year overall rate of survival after developing this cancer is still only 6,9% with a median survival of ≤ 15 months [2, 3]. The high mortality is mainly due to the rapidly recurs of this tumor and several factors contribute to it, such as a high intratumoral phenotypic heterogeneity and plasticity, as well as the feature of the hGBM cells to rapidly migrate and infiltrate within brain tissue, preventing a complete removal and inducing resistance to chemotherapy and radiotherapy. It follows that the residual cells develop alternative evolutionary paths that drive the growth of recurrent tumors and contribute to the treatment failure [4–6].

In order to identify the molecular processes underlying heterogeneity and plasticity of GBM cells, a bioinformatic analysis of the gene expression profile was initially performed by the Cancer Genome Atlas Consortium (TCGA), allowing to define the existence of four molecular clusters: proneural (PN), classical (CL), mesenchymal (MS) and neural (NE). The latter was excluded as it was contaminated with normal neural tissue [7, 8]. These subtypes of GBM identified by distinct molecular profiles are described to differ in their clinical courses and drug responses, being responsible for the failure of multimodal therapies, including RT, chemotherapy and other targeted therapies [7, 9–17].

Subsequently, Single-cell RNA-sequencing (scRNA-seq) studies have shown that several subtypes may exist simultaneously in different region of the same tumor and that molecular clusters can change over time and through therapy [8, 18, 19]. The combination of Single-cell seq and TCGA analysis, demonstrates that in GBM exist 4 cellular states that are reminiscent of canonical neurodevelopmental cell types: 1) neural-progenitor-like (NPC-like); 2) oligodendrocyte-progenitor-like (OPC-like); 3) astrocyte-like (AC-like), and 4) mesenchymal-like (MES-like). Each state or the combination of two of them is consistent with the three molecular subtypes previously described by TGCA. Actually, TGCA-CL and TCGA-MS correspond to AC-like and MES-like state, while TCGA-PN to OPC-like and NPC-like ones. As a result, each tumor turns out to be composed of cells that are in multiple cellular states and the tumor microenvironment could affect the transition among these different conditions or proliferation [20]. Although the identification of molecular subtypes has been of extreme importance by itself in understanding the molecular heterogeneity of the GBM, the source of a functional heterogeneity remains unclear and has had almost no translational impact on the clinical context and development of specific therapies.

The search for innovative and more effective therapies for GBM has benefited from the discovery that a relatively rare type of cancer cell, which possesses the cardinal features of an aberrant neural stem cell (NSCs), lie at the root of GBM insurgency propagation and perpetuation in humans, the glioblastoma stem cells (GSCs). This subpopulation of cells, endowed with tumor-propagating ability, express embryonic or tissue stem cell genes and, consistent with its role in supporting the relapse after therapy, is reported to be resistant to existing standard therapies [18, 21–27]. Importantly, GSCs cells have also been recently described to play a key role in underlying the phenotypic diversity and plasticity of GBM, which is strongly influenced also by the microenvironment [27–29]. Within the complexity of intratumoral heterogeneity, Richards and colleagues outlined that, down to the transcriptional and genetic level, GSCs can be dynamically retrieved in a combination of two phenotypic states (conditions), reproducing both neurodevelopmental and inflammatory functional programs. The switch from one program to another can occur based on early somatic alteration and copy number variation (CNV) state of GSCs and, in part, by cytokine signaling [29]. Patient-derived GSCs have also been described by different transcriptional profiles [8, 30, 31] and, importantly, to inherently display distinct level of stem cell markers depending on their GBM cluster of origin [32, 33]. Nevertheless, the intrinsic GSCs different subsets within the tumor itself and whether these differences might account for the functional inter-cluster variances in GBM subgroups are still under-investigated. Thus, GSCs remain a crucial elusive and difficult cellular target in glioblastoma treatment [6, 34].

By means of a standardized approach faithfully modeling GBM based on a cell system of stable, fully characterized GSCs lines, here we report that each transcriptional cluster of GBM patients contain different GSCs subpopulations endowed with distinctive molecular, functional and antigenic phenotypes, which are closely related to inherent different states of stemness. This allow the identification of new subtype-associated functional biomarkers predicting GBM patient stratification and survival, thus opening new and exciting possibilities for patient-tailored diagnostic and prognostic purposes, and, importantly, for the definition of individual patient-specific, drug-responsiveness therapeutic protocols by tackling different subset of GSCs.

## Methods

### Sample cohort features and population analysis

The sample cohort analyzed in this study was composed of 93 glioblastoma post-surgery tissues from patients who underwent neurosurgical resection at IRCCS National Neurologic Institute “C. Besta” and 34 GSCs lines established from them together with patients’ clinical data and matched peripheral blood (available from 27 patients). Clinical and sequencing information (GBM subtype, overall survival, *IDH1*, *TERT* promoter and *EGFR* status) are provided in Table 1. Tumor samples were collected in accordance to the ethical guidelines of the 2013 Declaration of Helsinki after approval of the institutional ethic board (protocol n°02 and protocol n°61) with signed informed consent. Material was anonymized at the time of collection. All samples were from patients with a confirmed diagnosis of GBM by a pathologist and classified according to the WHO guidelines. GSCs cells population, clonogenic and differentiation analyses were performed as in [22, 32]. The authenticity of each cell line was last checked in January 2021 by CNV profiling.

**Table 1:** Clinical characteristics of patients included in this study

ID Sample	SampleType^a^	Subtype	Sex	Age	Clinic.Censor	Clinic.OS_days	Clinic.IDH1	EGFRvIII	TERT Promoter	Clinic.Pathology
GBM#28	*TS*	PRONEURAL	M	69		DEATH	WT	NO	C228T	GBM
GBM#40	*TS/NS*	PRONEURAL	F	55		DEATH	WT	NO	C228T	GBM
GBM#21	*TS*	PRONEURAL	M	50		DEATH	WT	NO	WT	GBM
GBM#22	*TS*	PRONEURAL	M	61		DEATH	WT	NO	C228T	GBM
GBM#46	*TS/NS*	PRONEURAL	F	56		DEATH	WT	NO	C250T	GBM
GBM#38	*TS*	PRONEURAL	F	61		DEATH	WT	YES	C250T	GBM
GBM#39	*TS*	PRONEURAL	M	60		DEATH	WT	NO	WT	GBM
GBM#49	*TS/NS*	PRONEURAL	F	62		DEATH	WT	NO	C228T	GBM
GBM#62	*TS/NS*	PRONEURAL	M	70		DEATH	WT	NO	C228T	GBM
GBM#52	*TS/NS*	PRONEURAL	F	72		DEATH	WT	YES	C228T	GBM
GBM#56	*TS/NS*	PRONEURAL	M	61		DEATH	WT	NO	C228T	GBM
GBM#3	*NS*	PRONEURAL	M	73		DEATH	WT	NO	C228T	GBM
GBM#2	*NS*	PRONEURAL	M	61		DEATH	WT	NO	C228T	GBM
GBM#20	*TS*	PRONEURAL	F	83			WT	NO		GBM
GBM#93	*NS*	PRONEURAL	M	50		DEATH	WT	NO	WT	GBM
GBM#6	*TS/NS*	CLASSICAL	M	60			WT	NO	C228T	GBM
GBM#32	*TS*	CLASSICAL	M	43		DEATH	WT	YES	WT	GBM
GBM#1	*NS*	CLASSICAL	M	78		DEATH	WT	NO	C228T	GBM
GBM#45	*NS*	CLASSICAL	M	72			WT	NO	C228T	GBM
GBM#8	*TS*	CLASSICAL	F	63		DEATH	WT	YES	C228T	GBM
GBM#11	*TS*	CLASSICAL	M	63		DEATH	WT	YES	C228T	GBM
GBM#10	*TS*	CLASSICAL	M	75		DEATH	WT	NO	WT	GBM
GBM#25	*TS*	CLASSICAL	F	63			WT	YES	C228T	GBM
GBM#17	*TS*	CLASSICAL	M	53		DEATH	WT	NO	C228T	GBM
GBM#34	*TS*	CLASSICAL	F	74		DEATH	WT	NO	C250T	GBM
GBM#7	*TS*	CLASSICAL	F	47			WT	YES	C228T	GBM
GBM#12	*TS*	CLASSICAL	M	42		DEATH	WT	NO	C228T	GBM
GBM#13	*TS*	CLASSICAL	M	42		DEATH	WT	YES	C250T	GBM
GBM#29	*TS*	CLASSICAL	F	62		ALIVE	WT	NO	C228T	GBM
GBM#14	*TS*	CLASSICAL	M	77		DEATH	WT	YES	C228T	GBM
GBM#30	*TS*	CLASSICAL	M	52		DEATH	WT	NO	C228T	GBM
GBM#19	*TS*	CLASSICAL	F	56		DEATH	WT	YES	C228T	GBM
GBM#35	*TS*	CLASSICAL	M	67		DEATH	WT	NO	C228T	GBM
GBM#36	*TS*	CLASSICAL	M	72		DEATH	WT	NO	C228T	GBM
GBM#47	*TS/NS*	CLASSICAL	M	79		DEATH	WT	NO	C228T	GBM
GBM#58	*TS/NS*	CLASSICAL	F	63		DEATH	WT	YES	C228T	GBM
GBM#60	*TS/NS*	CLASSICAL	M	54		ALIVE	WT	YES	C228T	GBM
GBM#61	*TS/NS*	CLASSICAL	F	75		DEATH	WT	YES	C228T	GBM
GBM#59	*TS/NS*	CLASSICAL	F	59		DEATH	WT	NO	WT	GBM
GBM#50	*TS/NS*	CLASSICAL	M	76		DEATH	WT	YES	C228T	GBM
GBM#67	*TS/NS*	CLASSICAL	M	44		DEATH	WT	YES	C250T	GBM
GBM#27	*TS*	CLASSICAL	F	69			WT	NO		GBM
GBM#51	*TS/NS*	CLASSICAL	M	79		ALIVE	WT	YES	C228T	GBM
GBM#65	*TS/NS*	CLASSICAL	M	45		DEATH	WT	NO	WT	GBM
GBM#64	*TS/NS*	CLASSICAL	F	46		DEATH	WT	YES	C228T	GBM
GBM#66	*TS/NS*	CLASSICAL	F	44		DEATH	WT	NO	C250T	GBM
GBM#4	*NS*	CLASSICAL	F	79		DEATH	WT	NO	C228T	GBM
GBM#16	*TS*	CLASSICAL	M	58		DEATH	WT	NO	C250T	GBM GIANT
GBM#9	*TS*	CLASSICAL	F	65			WT	YES	C228T	GBM
GBM#70	*TS*	CLASSICAL	M	79		DEATH	WT	YES	C228T	GBM
GBM#71	*TS*	CLASSICAL	F	37		DEATH	WT	YES	WT	GBM
GBM#72	*TS*	CLASSICAL	M	74		DEATH	WT	NO	C228T	GBM
GBM#74	*TS*	CLASSICAL	F	64		DEATH	WT	YES	WT	GBM
GBM#79	*TS*	CLASSICAL	M	43		DEATH	WT	YES	C228T	GBM
GBM#80	*TS*	CLASSICAL	M	41		ALIVE	WT	YES	C228T	GBM
GBM#81	*TS*	CLASSICAL	M	75		DEATH	WT	NO	C250T	GBM
GBM#83	*TS*	CLASSICAL	M	39		DEATH	WT	NO	C250T	GBM
GBM#84	*TS*	CLASSICAL	M	50		DEATH	WT	YES	C228T	GBM
GBM#85	*TS*	CLASSICAL	M	53		DEATH	WT	YES	WT	GBM
GBM#87	*TS*	CLASSICAL	M	57		DEATH	WT	NO	C228T	GBM
GBM#88	*TS*	CLASSICAL	F	58		DEATH	WT	YES	C228T	GBM
GBM#89	*TS*	CLASSICAL	M	67		DEATH	WT	YES	C228T	GBM
GBM#91	*TS*	CLASSICAL	M	67		DEATH	WT	YES	C250T	GBM
GBM#92	*TS*	CLASSICAL	M	66		DEATH	WT	NO	C228T	GBM
GBM#31	*TS*	MESENCHYMAL	F	69		DEATH	WT	YES	C228T	GBM
GBM#33	*TS*	MESENCHYMAL	M	77		DEATH	WT	NO	C250T	GBM
GBM#23	*TS*	MESENCHYMAL	M	72		DEATH	WT	NO	C250T	GBM
GBM#41	*TS/NS*	MESENCHYMAL	F	77		DEATH	WT	NO	C228T	GBM
GBM#26	*TS*	MESENCHYMAL	F	63			WT	NO	C250T	GBM
GBM#15	*TS*	MESENCHYMAL	M	62		DEATH	WT	NO	WT	GBM GIANT
GBM#42	*TS/NS*	MESENCHYMAL	F	51		DEATH	WT	NO	C250T	GBM
GBM#24	*TS*	MESENCHYMAL	M	41		DEATH	WT	NO	C250T	GBM
GBM#18	*TS*	MESENCHYMAL	M	60		DEATH	WT	NO	C228T	GBM
GBM#43	*TS/NS*	MESENCHYMAL	F	43		DEATH	WT	NO	C228T	GLIOSARCOMA
GBM#44	*TS/NS*	MESENCHYMAL	M	10		DEATH	WT	NO	WT	GBM GIANT
GBM#37	*TS*	MESENCHYMAL	M	64		DEATH	WT	NO	C228T	GBM
GBM#48	*TS/NS*	MESENCHYMAL	M	38		DEATH	WT	NO	C250T	GBM
GBM#63	*TS/NS*	MESENCHYMAL	M	50		DEATH	WT	NO	C228T	GBM
GBM#53	*TS/NS*	MESENCHYMAL	M	70		DEATH	WT	NO	C228T	GBM
GBM#54	*TS/NS*	MESENCHYMAL	M	41		DEATH	WT	NO	C228T	GBM
GBM#55	*TS/NS*	MESENCHYMAL	M	55		DEATH	WT	NO	C228T	GBM
GBM#57	*TS/NS*	MESENCHYMAL	M	69		DEATH	WT	NO	C228T	GBM
GBM#5	*TS/MS*	MESENCHYMAL	F	71		DEATH	WT	NO	C250T	GBM
GBM#90	*TS*	MESENCHYMAL	M	56		DEATH	WT	NO	C228T	GBM
GBM#68	*TS*	MESENCHYMAL	M	50		DEATH	WT	NO	C228T	GBM
GBM#69	*TS*	MESENCHYMAL	F	72		DEATH	WT	NO	C228T	GBM
GBM#73	*TS*	MESENCHYMAL	M	80		DEATH	WT	NO	C228T	GBM
GBM#75	*TS*	MESENCHYMAL	M	65		ALIVE	MUT	NO	WT	GBM GIANT
GBM#76	*TS*	MESENCHYMAL	M	63		DEATH	WT	NO	WT	GBM
GBM#77	*TS*	MESENCHYMAL	F	69		DEATH	WT	NO	C228T	GBM
GBM#78	*TS*	MESENCHYMAL	M	51		DEATH	WT	NO	C228T	GBM
GBM#82	*TS*	MESENCHYMAL	F	48		DEATH	WT	NO	C228T	GBM
GBM#86	*TS*	MESENCHYMAL	M	55		DEATH	WT	NO	C228T	GBM

^a^*TS* Tissue and *NS* Neurosphere

### In vivo studies

All animal analyses were performed according to the Guidelines for the Care and Use of Laboratory Animals and experimental protocols approved by the Italian Ministry of Health (805/2016-PR and 119/2019-PR). Anesthesia and analgesics were used in order to minimize any suffering of the animals. Tumorigenic capacity of GSCs lines and their in vivo phenotypic hallmarks were examined by stereotactic injection of 3 × 10^5 firefly luciferase-transduced (F-luc) GSCs cells from different subtypes into the right striatum of SCID mice (Charles River Lab) as previously described [22, 32, 33, 35]. Mice were checked daily for any signs of distress and monitored weekly by ventral and dorsal views with In vivo Lumina (Xenogen, PerkinElmer Inc) and tumor growth was indirectly quantified. Mice were then sacrificed at different endpoints as soon as they became symptomatic according to the subtype of the GSCs injected, and their brains were collected after transcardiac perfusion and processed as previously described [22, 32, 33, 35, 36].

### Survival analysis

To evaluate the relationship between the level of *EGFR* and *DCX* and patients’ outcome, 117 IDH1 wild-type GBM patients were selected in the TCGA dataset [37] and mRNA expression data with corresponding clinical information downloaded from https://xenabrowser.net/datapages/. Optimal cutoff between high and low mRNA expression groups were determined through the R package “survminer”. 83 high-grade glioma patients of our cohort with available clinical follow up data were stratified into TCGA subtypes (*n* = 13 TCGA-PN, *n* = 28 TCGA-MS and *n* = 42 TCGA-CL) and their survival data analyzed with Kaplan–Meier plot by GraphPad Prism v.7.0 software. Overall comparisons were performed by Log-rank test. *P*-values < 0.05 were considered significant.

### Statistical analyses

In vitro data were analyzed using R and GraphPad Prism v7.0 software with statistical test selected according to the variance and distribution of data. Gene expression analysis and differences among groups originated from microarray sequencing was performed by ANOVA test with a *q*-value < 0.05 considered significant. Sequencing and SNPs arrays data were examined by comparing each cluster to the others with a two-tailed Fisher’s exact test. Canonical pathway enrichment analysis on somatic variants was performed with Pathscore [38], which is based on a collection of ‘canonical pathways’ from the Molecular Signatures Database [39] including pathways from the KEGG, Biocarta, Reactome and Nature-NCI databases. Analysis of the biological functions of differently altered gene copy number and differently expressed mRNAs was performed by Ingenuity Pathway Analysis (IPA; Qiagen, http://www.ingenuity.com/) and R software [32, 33, 35, 40] with a right-tailed Fischer’s exact test. *P*-values were further adjusted and cutoff for significance was set as *q*-values < 0.05 and activating z-score threshold ≥ 2 or inhibiting z-score threshold ≤ 2.

To assess cell stability, CNV mean values at different time points were compared. According to their difference, we classified as amplification a difference larger than 0.5 and as deletion a difference smaller than 0.5. Therefore, we cross-tabulated the amplification/deletion status at two different time points in order to assess cell stability. As observed for each cell line, changes in copy number status (i.e. from amplification to deletion or from deletion to amplification) were observed for a very small and negligible number of genes (< 5%). To assess and compared trend over time, in vitro GSCs growth curves and in vivo GSCs-tumors growth rate across GBM clusters was analyzed with hierarchical linear models for repeated measurements by SAS Statistical Package Release 9.4 (SAS Institute) [41, 42]. As outcome, the log-transformed number of cell number. Unequally spaced time occasions within the experiments were counted by a spatial power correlation type [41]. A 5% cutoff was applied to validate data significance. Overall survival of GSCs-implanted mice among GBM subtypes was examined with GraphPad Prism v7.0 software by Kaplan-Meyer curves based on survival algorithms determined by the log rank Mantel-Cox and Gehan-Breslow Wilcoxon tests. Groups were compared by respective median survival of number of days taken to reach 50% morbidity. *P* < 0.05 as significant.

## Results

### Molecular hallmarks of GBM patients and their sibling GSCs lines across TCGA clusters

**Fig. 1:**
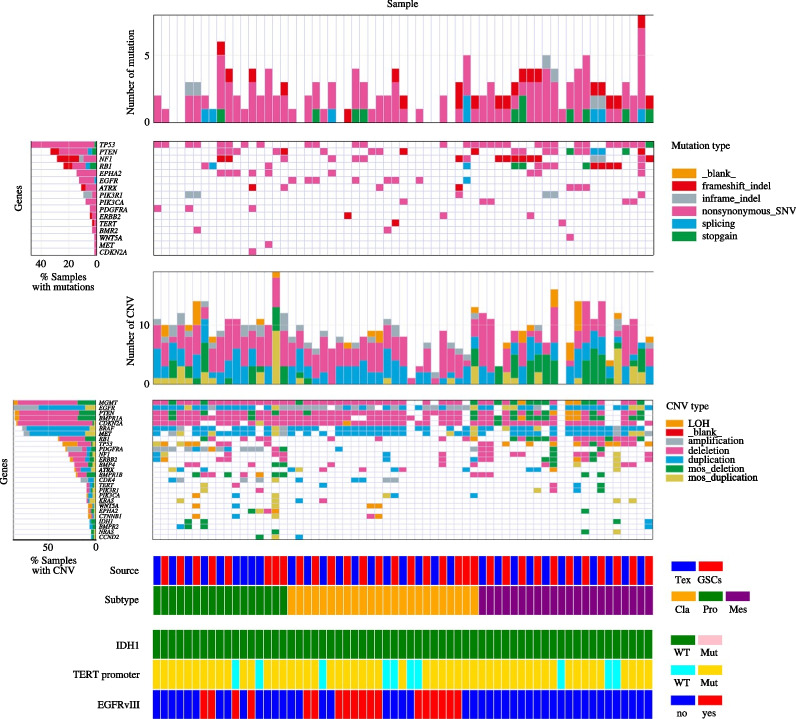
Comprehensive characterization of GBM patients and their sibling GSCs lines. Integrated matrix of tissue samples from grade IV glioma patients (Tex; blue) and GSCs lines (GSCs; red) across subtypes reporting gene variants (indels, SNVs, splicing, stopgain) together with significant CNVs and allelic frequencies (loss of heterozygosity; LOH, amplification, deletion, duplication, mosaicisms). For each single sample, columns represent number and type of genetic alterations. Genetic variations are reported according to their frequency in GBM tissues and their GSCs (% samples with mutations and % samples with CNVs). The status of *IDH1* and *TERT* promoter genes and the presence of *EGFRvIII* is also depicted

**Fig. 2:**
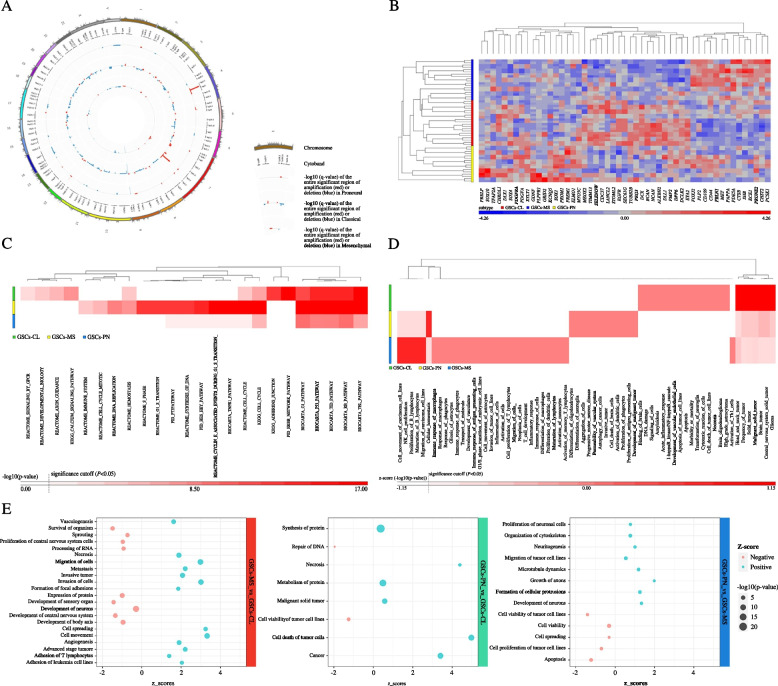
Genetic and transcriptional phenotypes of GBM patients and their GSCs. **A** Circos plot reporting genomic copy number variation (CNV) profiling, and the relative cytobands involved, for high-grade tissues and their derivative GSCs displaying the differences retrieved in alteration frequency among subtypes, as emerged from GISTIC analysis. In each case, the outer track provides alterations in proneural samples, inside the circle those of classical cases and the inner track shows variations shared by all the mesenchymal samples. -log10(*q*-value) of the significant region of amplification or deletion is reported in red or blue, respectively. **B** Heat map of one-way hierarchical clustering of 51 differentially enriched genes in GSCs lines clearly outlining three distinctive clustering in an unsupervised manner. A dual-color code represents genes up- (red) and down-regulated (blue), respectively. **C** Hierarchical clustering analysis using 24 over-represented altered pathways in GBM patients and their GSCs reporting that TCGA-MS cases mainly displayed aberrations in immune system, DNA replication and TNFR1 pathways, whereas TCGA-CL ones in calcium signaling, ERBB network signaling, necrosis and apoptosis functions. TCGA-PN samples were instead mainly characterized by aberrant cellular homeostasis, differentiation of oligodendrocytes and neuroglia, activation of dendritic cells pathways. GBM subtypes are coded by color. Pathways are colored in shades of red according to the different level of significance. **D** Unsupervised hierarchical clustering using 66 significant differently enriched biological processes associated to gained or lost genes in GBM patients and their derivative GSCs lines across subtypes, as emerged from IPA analysis. GBM subclusters are coded by color. Pathways are colored in shades of red according to the different level of significance. **E** Bubble plots showing that over-represented genes and their biological process ranked by FDR in TCGA-MS GSCs are predominantly related to *cell movement* and *migration*, whereas those over-expressed in TCGA-CL and TCGA-PN cells are mostly entailed with *cell proliferation* and *neurogenesis*, respectively. The enrichment in each cluster is relative to one another. The size of the circles represents -log_10_ (*P*-value) while the colors correspond to positive (blue) or negative (red) z-score

To get insight into the distinct aberrant molecular signature differentiating GBM subtypes [7, 20, 37, 43], high-grade glioma tissues, matched blood DNA and their derivative GSCs lines were sequenced and then analyzed for their expression program. As shown by the integrated matrix in Fig. 1 and Supplementary Fig. 1, we found that each GBM patient together with the cognate GSCs line displayed a distinctive landscape of somatic mutations and copy number alterations across GBM clusters. Mutation-calling analysis revealed a comparable number of somatic variants between GBM patients and their GSCs, whereas significant differences emerged when the three molecular clusters were compared to each other (**P = 0.005, Kruskal–Wallis test) (Supplementary Fig. 2A and Supplementary Tables 1 and 2). Analysis of molecular abnormalities’ distribution confirmed that, among other genes, TCGA-CL samples predominantly harbored somatic mutations in *EGFR* gene (***P < 0.001, two-sided Fisher’s exact test), also reporting its constitutive variant *EGFRvIII,* known to confer enhanced tumorigenic behavior and pharmacological-resistance [11, 27] (***P < 0.001) (Fig. 1, Supplementary Fig. 2B, C and Supplementary Table 2). Chromosome (chr) 7 amplification, with focal amplification at 7p11.2 (*EGFR*), paired with chr10 loss, which mostly co-occurred (46%) with the constitutively active variant *EGFRvIII,* was also highlighted together with significant gains at 3p12.1 and 4p15.33 (*q* value = 0.02) (*RHOA*, *EPHB1* and *BMPR1B*, *CAMK2D*) (Fig. 2A and Supplementary Table 3). Consistently, classical cases were mostly highlighted by the highest level of *EGFR*, *NES*, *AQP4*, *CDKN2A*, *BCAN*, *DCLK1*, *VCAN* and *DLL1* (***P < 0.001, **P < 0.01, *P < 0.05, Kruskal–Wallis test) (Fig. 2B and Supplementary Fig. 2D-F). *PTEN* and *NF1* genes, usually reported as commuted, have been observed harboring the highest rate of mutation in mesenchymal cases, together with *RB1* and *PIK3CA* (***P < 0.001, **P < 0.01). In the same cluster, focal amplifications at 21q21.3 (*q* value = 0.03) (*TIAM1*, *ABCG1*, *OLIG1*) and 2p24.3 (*q* value = 0.07) (*MYCN*) were mainly reported together with an enrichment for genes associated with immune/complement responses and inflammation (*CD109*, *C1R, PAPPA, CTSB*) and underlining an invasive “mesenchymal” signature *(LOX*, *CHI3L1, MET*, *CD44)*. The highest rate of *PDGFRA* (*P < 0.05), *EPHA2* and *BMPR2* variations was retrieved in proneural cases, also mostly characterized by focal amplification at 4q12 (*q* value = 3.2881E-23) (*PDGFRA*), associated in the 29% of case with the amplification at 12q13.3, harboring *CDK4* gene [11], and 7p14.3 (*q* value = 0.02) (*DLX5*, *EPHB6*, *HOXA10*). Proneural patients and their GSCs were also found to predominantly express developmental genes (*SLITRK2, DLX1*, *PDGFRA*, *OLIG2, NKX2)* and the highest level of the known stemness-associated genes (*SOX11*, *DCX*, *HOXA7*, *EPHA2* and *PROM1)*. Remarkably, considerable overlap in genetic aberrations between pairs of GBM patient’s tissue and GSCs line was observed (Supplementary Fig. 3A), being the latter also reported genetically stable over time (Supplementary Table 4).

Gene enrichment analysis on SNVs and CNVs [44, 45] and functional analysis performed on the global gene expression profiles of GBM patients and their GSCs outlined the existence of distinct, cluster-related, biological damaged profiles and enrichment for gene sets (Fig. 1 and Supplementary Fig. 3B*).* Particularly, genetic alterations of *immune system*, DNA replication and *TNFR1* pathways occurred exclusively in mesenchymal cases, which also harbored aberrations involved in key biological processes as *immune response*, *migration and invasion of cells* and *inflammatory response* (*P* < 0.001, two-sided Fisher’s exact test, corrected for multiple testing using a Familywise Error Rate) (Fig. 1 and Supplementary Table 4). The same cluster was described by an expression pattern, which included genes regulating *invasion*, *metastasis*, *angiogenesis* and *activation of immune system* (Fig. 1, Supplementary Fig. 3B and Supplementary Tables 5 and 6). Conversely*, calcium signaling*, *adherens junction* and *axon guidance* pathways (*P* < 0.05), *ERBB network* signaling, *necrosis and apoptosis* functions were predominantly disrupted in classical patients, who also reported significant enhancements in the expression of selective signaling including *proliferation and cycling* and *cell viability*. *Cellular homeostasis*, *differentiation of oligodendrocytes and neuroglia*, *activation of dendritic cells*, *proliferation of phagocytes and antigen presenting cells* and *permeability of vascular system* functions were mainly targeted in proneural cases, identified by genes involved in *CNS development and function*, *neuritogenesis*, *tissue and cellular development*.

These findings underlined that peculiar pathogenic features discriminate each GBM patient and its sibling GSCs line across GBM clusters.

### Distinct functional pools of GSCs distinguish GBM patients across molecular subtypes

**Fig. 3:**
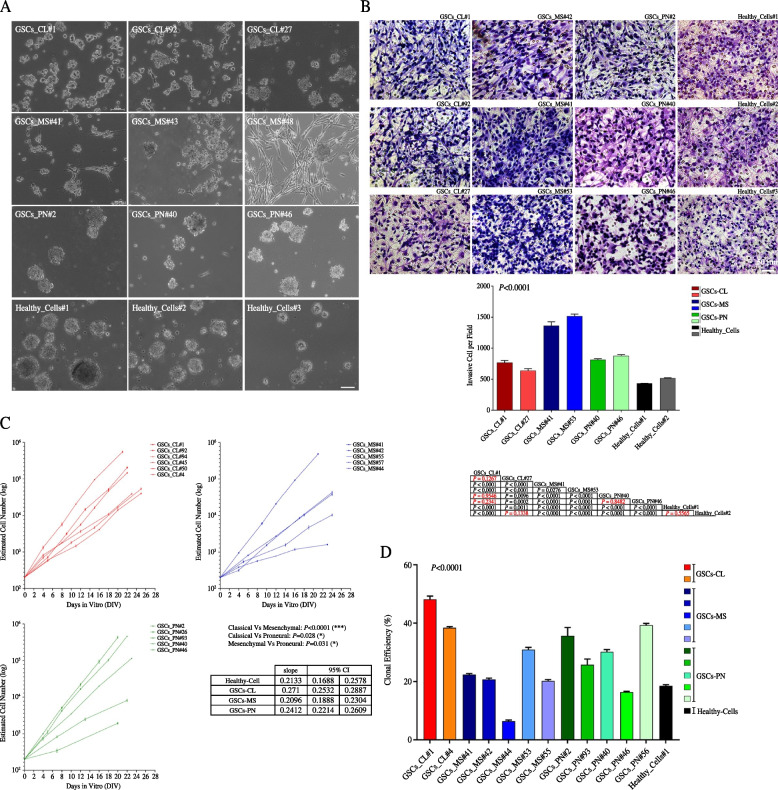
Distinct functional GSCs subsets might be discriminated within GBM subtypes. **A** Phase-bright microphotographs of neurospheres from classical, mesenchymal and proneural GSCs cultures and human neural stem cells (NSCs). Bar: 100 µm. **B** In vitro migration assays reporting the higher degree of invasiveness in TCGA-MS GSCs versus their TCGA-CL and PN counterpart and normal neural stem cells. *Bar*: 50 µm (Top). Quantification is shown as mean ± SEM (bottom). *P*-values from ANOVA multiple comparison test are reported. **C**-**D** Analysis of long-term (**C**) and short-term (**D**) proliferation showing that mesenchymal and proneural GSCs (blue and green lines, respectively) are identified by an inherent expansion rate and a self-renewal capacity significantly much lower than that one of classical GSCs (red lines) (hierarchical linear model for repeated measurements). Slope’s value and confidence interval (CI) are shown

We next tested as to whether the differential molecular profiles identified across GBM patients might be associated to an inherent different functional state of GSCs. As shown in Fig. 3A, the specific enhancement in the expression of proliferative signaling distinctive for classical patients was reinforced by the typical rounded morphology displayed by neurospheres obtained through the enrichment of cells isolated from the post-surgery specimen [22, 32, 33, 35]. When the tumor proliferation rate was measured at the protein level in either GBM tissues or in their derivative GSCs by counting the cells positive for the proliferation markers Ki67 and MELK, TCGA-CL samples were confirmed to propagate more extensively than tumors and cells from either TCGA-MS or TCGA-PN subtype (Supplementary Fig. 4A, B). Irregularly shaped clones, characterized by the presence of many protruding and elongated cells, suggestive of increased cell adhesion, were instead generated in GSCs cultures from TCGA-MS cases, enriched in the invasive/infiltrative and EMT signaling pathways. Tumors from these patients were also shown to be highly vascularized, with a total vascular area > threefold higher than in classical tumors, as measured by CD31 + cells (***P < 0.001, **P < 0.01, *P < 0.05, one-way ANOVA, *n* = 5) (Supplementary Fig. 4A, B). Meanwhile, GSCs from the TCGA-PN counterpart, pinpointed by high levels of signaling involved in neurogenesis and the highest expression of the markers of stemness EphA2 and SSEA1 [35, 46], displayed the typical regular morphology, being much similar to human normal neural stem cells (NSCs) (Fig. 3A and Supplementary Fig. 4A, B).

Next, we assessed whether the increased migratory and invasive phenotype of mesenchymal population might well represent a cell-autonomous trait of these cells by in vitro migration assays [32, 33, 47]. As clearly reported in Fig. 3B, mesenchymal GSCs were able to invade much more efficiently displaying a significant higher infiltrative phenotype as compared to that one of classical GSCs. The pattern displayed by proneural GSCs was similar to that one of mesenchymal GSCs. When GSCs’ global growth trend was compared across subtypes, the inherently expansion rate (Fig. 3C) and self-renewing capacity (Fig. 3D) of mesenchymal and proneural GSCs was shown to remain strikingly lower as compared to their classical counterpart, suggesting that GBM clusters might contain GSCs’ subpopulations intrinsically endowed with differential self-renewal ability (*P* < 0.0001 TCGA-CL vs. TCGA-MS and *P* = 0.028, TCGA-CL vs. TCGA-PN, hierarchical linear model for repeated measurements and ***P < 0.0001, ANOVA multiple comparison test, respectively) (Fig. 3C). Strikingly, confidence intervals emerged by cut-offs analysis suggested that growth slope between 0.2532 and 0.2887 should likely classify GSCs cells as classical, between 0.2214 and 0.2609 as proneural and between 0.1888 and 0.2304 as mesenchymal (Fig. 3C). Consistently, differentiated [22, 32, 33, 35] fast-growing GSCs from the TCGA-CL cluster were shown to comprise the highest fraction of abnormal cells which promiscuously co-expressed the astroglial marker GFAP with the neuronal marker Tuj1 [22, 48] as compared to the terminally differentiated mesenchymal and proneural cultures, which displayed a more “mature”, GFAP-positive astroglial phenotype (***P < 0.0001,**P < 0.001,*P < 0.05, Kruskal–Wallis ANOVA, dunn’s test) (Supplementary Fig. 4C).

Findings so far lent to the conclusion that distinct subsets of GSCs endowed with differential state of stemness inherently exist within GBM molecular clusters and might distinguish their classification.

### A distinct tumorigenic potential identifies GSCs subpopulation across subtypes

**Fig. 4:**
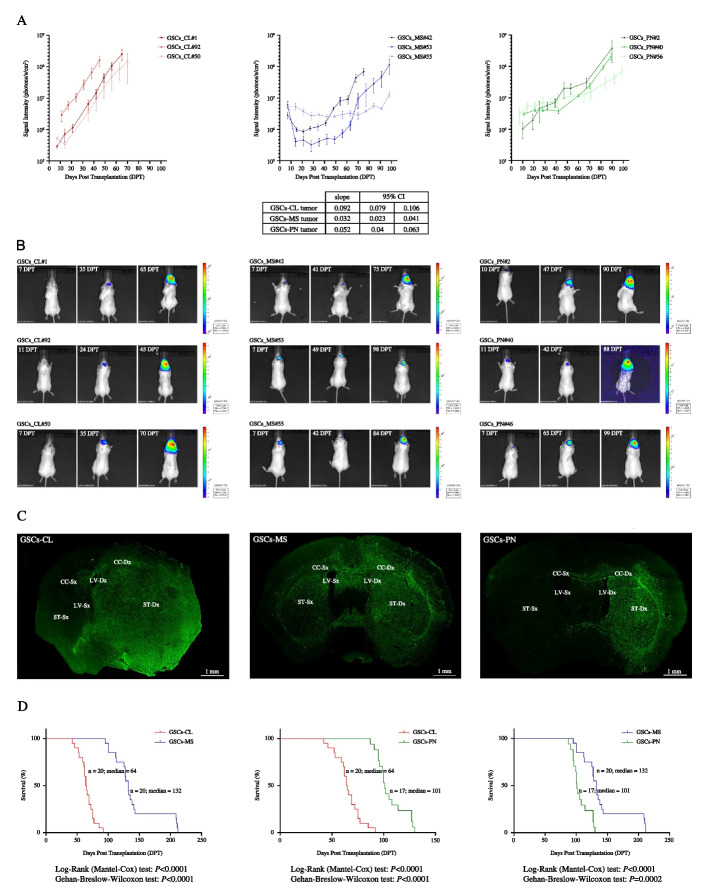
GSCs populations are inherently endowed with different tumorigenic and lethal potentials. **A** Quantitative time-course imaging analysis demonstrating that GBM xenografts from classical (shades of red lines; left), mesenchymal (shades of blue lines; middle) and proneural (shades of green lines; right) GSCs display heterogeneous tumor growth with the former being faster than the others. Error bars indicate mean ± SEM. Slope’s value and CI are shown. **B** Imaging of luciferase-tagged TCGA-CL (left), MS (middle) and TCGA-PN (right) GSCs injected into the brain of *SCID* mice from 7–10 DPT next to the end-stage disease typical of each subtype injected. **C** Immunohistochemical reconstruction analysis of brain samples from the experiment in **A** demonstrating that spreading of those tumors established by mesenchymal and proneural GSCs, with cells that infiltrated extensively from the transplantation site within the contralateral hemisphere is significantly enhanced as compared to classical GSCs-tumors, which display more compact masses. (ST: striatum; CC: corpus callosum; LV: lateral ventricle). *Bars*, 100 *u*m and 1 mm. **D** Kaplan–Meier plots of overall survival showing that mice transplanted with mesenchymal GSCs (*n* = 20; blue lines; median = 132 days) survived longer than those implanted with classical (*n* = 20; red lines; median = 64 days) and proneural (*n* = 17; green lines; median = 101 days) lines. *P*-values from Log-rank and Gehan Breslow-Wilcoxon tests are shown

To finally assess a functional association between the different biological hallmarks of GBM subtypes and an inherent enrichment of a specific GSCs subpopulation, we generated GBM xenografts by transplanting GSCs into the brain of immunocompromised *SCID* mice. As expected, consistent with the in vitro observations, upon orthotopic injection of distinct sets of GSCs from different subtypes, interesting differential tumorigenic and lethal potentials were observed (Fig. 4). More specifically, classical GSCs established tumors which were fast growing (*P* < 0.001 vs. TCGA-PN and TCGA-MS, hierarchical linear models for repeated measurements) but less invasive as compared to proneural (*P* = 0.009 vs. TCGA-MS) and mesenchymal ones (Fig. 4A, B). Furthermore, confidence intervals emerged from the comparisons of growth rates indicated that tumors displaying a slope between 0.079 and 0.106 should be likely classified as classical, between 0.040 and 0.063 as proneural and between 0.023 and 0.041 as mesenchymal (Fig. 4A).

Remarkably, as confirmed by serial reconstruction performed at the histological and stereological level in Fig. 4C, the xenografting of classical GSCs established more extended and delimitated tumor masses as compared to those of mesenchymal GSCs, which gave rise to intracranial tumors with a strikingly faster and broader infiltration pattern. Proneural GBM generated upon intracranial transplantation, stood in the middle of this spectrum. Consistently, analysis of overall survival reported that mice receiving classical GSCs died significantly earlier (*n* = 20 mice; median = 64 days) than those xenografted with mesenchymal (*n* = 20 mice; median = 132 days) and proneural (*n* = 17 mice; median = 101 days) cells (*P* < 0.001, Log-rank test) (Fig. 4D).

All of these data confirmed that distinct GSCs populations might discriminate GBM patients’ phenotype across molecular clusters.

### Classification and prediction of GBM patients by means of stemness-related markers

**Fig. 5:**
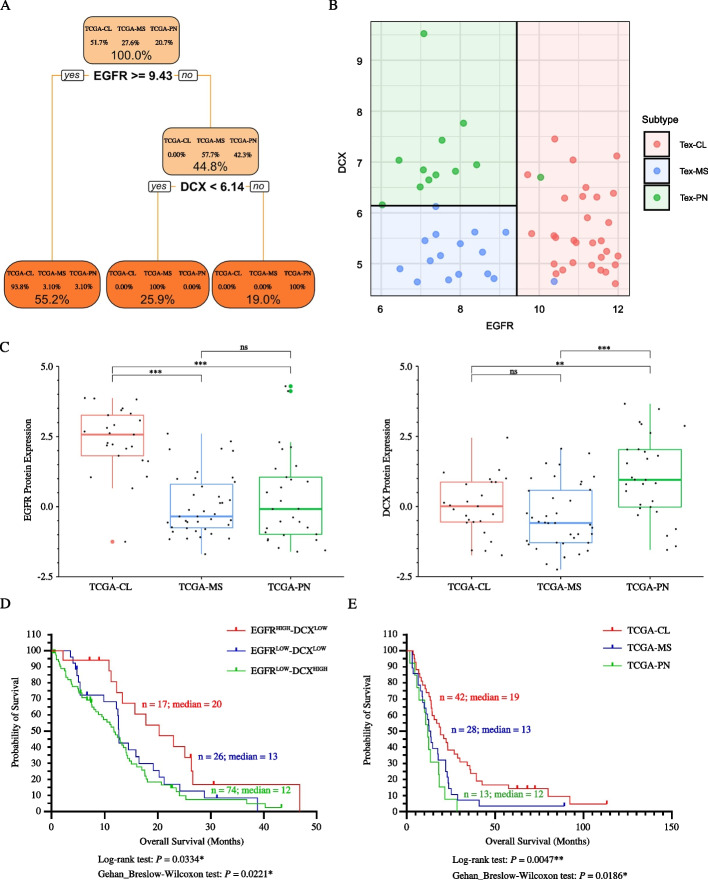
EGFR and DCX levels discriminate GBM patients’ subtype and survival. **A** Classification tree from recursive portioning tree analysis reporting the splitting variables between branches along with their optimal cut-offs in terms of log2 expression. The final leaves (in orange) report the percentages of each tissue classes (within the leaf) and the percentage of the total number of tissues that fall in this leaf (between leaves). **B** Plot depicting how patient’s tissues are classified into the three classes (final leaves of the classification tree). **C** *DCX* and *EGFR* differential protein expression analysis from [53] dataset across GBM subtypes. The visualization of proteins abundance is reported as box plots with median ± IQR. ***P < 0.001, ** *P* < 0.01, * *P* < 0.05, Mann–Whitney U test, two-tailed. **D** Kaplan–Meier curves of overall survival showing that, according to our dataset, GBM patients with an *EGFR*^High^-*DCX*^Low^ profile (blue line) died earlier than those with *EGFR*^Low^-*DCX*^High^ and *EGFR*^Low^-*DCX*^Low^ fingerprint (green and red lines, respectively). Mantel-Cox and Breslow-Wilcoxon test, log-rank *P*-value = 0.033 and *P*-value = 0.022, respectively. **E** Kaplan–Meier plot of overall survival showing that classical (*n* = 42; blue line) GBM patients survived longer than mesenchymal (*n* = 28; red line) and proneural (*n* = 13; green line) ones. Mantel-Cox and Breslow-Wilcoxon test, log-rank *P*-value = 0.047 and *P*-value = 0.017, respectively

Having perceived in GBM molecular subtypes an association between a distinct biological profile and an inherent enrichment of different GSCs pools, we finally tested the combination of two biomarkers emerged selectively represented for their ability to discriminate and predict GBM clusters. Among the candidates described (Fig. 2B and Supplementary Fig. 2D-F), classification tree analysis revealed that three heterogeneous expression of doublecortin (*DCX*) and *EGFR* genes, the former associated with neurodevelopmental and progenitor cell signatures and the latter with stemness [20, 49–52], almost perfectly segregated GBM patients. In details, patients showing an *EGFR* expression larger or equal to 9.43 were very likely (93.8%) allocated to the classical cluster. On the other hand, cases described by an *EGFR* expression lower than 9.43 and, concomitantly, a *DCX* expression lower than 6.14 belonged were included without error (100%) within the mesenchymal subtype class. Finally, tissues showing *EGFR* level lower than 9.43 and, concomitantly, a *DCX* expression larger or equal to 6.14 were surely (100%) characterized by a proneural fingerprint (Fig. 5A). Remarkably, plots in Fig. 5B reinforced the correlation between the inherent complementary regulation of these biomarkers and patient’s TCGA cluster of origin, revealing tissues from the classical subtype identified by an EGFR^High^-DCX^Low^ profile, whereas proneural GBM by a complementary EGFR^Low^-DCX^High^ percentage. Mesenchymal cases accounted for the EGFR^Low^-DCX^Low^ subpopulation. These results were further confirmed when analyzing EGFR and DCX protein expression in the public dataset [53]. Among GBM subtypes, the highest EGFR level was shown to be distinctive of the classical cluster, whereas DCX protein of the proneural group (Fig. 5C). As shown by Kaplan–Meier plots in Fig. 5D, when analyzing and comparing the outcome of patients defined by the three different patterns of *DCX* and *EGFR* expression according to our dataset, cases described by low *EGFR* and, concomitantly, high *DCX* level died earlier (*n* = 74; median = 12 months) than those with an EGFR^Low^-DCX^Low^ profile (n = 26; median = 13 months) and those depicting high *EGFR* and low *DCX* expression, who was the one with the longest survival (n = 17; median = 20 months)(*P* = 0.0334, Log-rank test and *P* = 0.0221, Gehan-Breslow-Wilcoxon tests). Remarkably, this was well in agreement with survival curves of GBM patients across subtypes (*n* = 83 *IDH1* wild-type GBM patients), in which proneural cases was shown to exhibit more lethal tumors (*n* = 13; median = 12 months), than the mesenchymal (*n* = 28; median = 13 months) and classical ones (*n* = 42; median = 19 months)(*P* = 0.0047, Log-rank test and *P* = 0.0186, Gehan-Breslow-Wilcoxon test) (Fig. 5E).

Data here demonstrate that stemness-related markers differently enriched among GSCs subsets can discriminate and predict the molecular subtype of GBM patients, representing new opportunities for patient-tailored diagnostic and prognostic purposes.

## Discussion

In the last decade, high-throughput profiling analysis have helped decipher glioblastoma heterogeneous molecular profile, outcome and therapeutic response (7–9; 17–20). This lent to the concept of developing molecularly targeted therapies, to provide personalized treatments that are also more effective and less toxic than conventional chemotherapy [54, 55]. Thus, new therapeutics approaches have recently been focused on tackling a number of signaling pathways implicated in GBM development, like those related to intrinsic survival, cell cycle and apoptosis or metabolism, and intrinsically altered molecules [54, 56, 57]. While this approach promises to significantly improve the therapeutic scenario, the development of new markers, the identification of specific molecular targets and the overall process of developing therapeutics for GBM has been hampered by the lack of information as to the actual identity and nature of the normal cell type(s) that were hit by transformation and more importantly by the absence in a detailed understanding of the heterogeneity that exists within this tumor and a fundamental understanding of the cell types that are involved in tumor promotion and relapse. The discovery that GBMs embody cells endowed with tumor-initiating ability and all of the functional features that define stem cells of the CNS, the so-called glioblastoma stem cells (GSCs), has opened new frontiers for the development of potential new therapeutic approaches for this tumor. Major efforts are now directed at targeting GSCs for new therapeutic purposes, endowed with high and selective toxicity towards the tumor but innocuous towards normal cells [58–60].

In this context, here, we isolated, established and characterized [22, 32, 33, 35] a large enough culture collection of functionally stable GSCs, collected and studied their surgery tissues of origin (grade IV glioma). In considering GBM heterogeneity and its recent stratification in different subtypes and cellular programs [7, 20, 37, 43], each single GSCs primary line has been subjected to a comprehensive differential analysis for their molecular and antigenic profile, in comparison with the cognate tissue of origin, to each-other and to normal neural stem cells (NSCs) and fully characterized for their stem-like and tumor-initiating characteristics. Here we described that distinct and unique molecular, functional and antigenic profiles of GSCs cells, particularly with regard to stem cells properties and expression of novel critical regulators, were related not only to the transcriptional cluster of their GBM patient of origin but, strikingly, to inherently different states of stemness. In agreement with our previous studies [32, 33], here we found that classical GSCs pool was more undifferentiated and endowed with enhanced expansion capacity as compared to the more mature, relatively slow-propagating proneural and mesenchymal cells, likely endowed with a higher potential for infiltration.

We first identified critical aberrant pathways and networks specific to the distinct GSCs molecular phenotypes. We found that TCGA-MS GBM tissues and their sibling GSCs were defined by highly invasive genetic and mutational programs, which included mediators of tissue invasiveness and angiogenesis, cytoskeleton rearrangements, markers involved in the extracellular communication and cell migration and invasion with a typical invasive signature. TCGA-CL specimens were regulated by effectors included in genomic abnormalities and biological pathways controlling cell growth, proliferation and cycling, while TCGA-PN tissues and their GSCs were defined by CNS development and function, tissue and cellular development mutational and biological patterns and the highest levels of the known “stemness” markers (Fig. 2 and Supplementary Figs. 1–4). Notably, in agreement with our previous studies [22, 32, 35] when comparing side-by-side GBM specimens and sibling GSCs lines, we found that the latter faithfully fully resembled the main aberrant physio-pathological and phenotypic features of their tissue and, thus, of their patient of origin (Supplementary Fig. 3A), suggesting once more the usefulness of GSCs-tailored anti-GBM strategies instead of the “one fits all” GBM therapeutic approaches.

We confirmed the whole molecular features when functionally fully characterizing in vitro GSCs’ fingerprint and physio-pathological hallmarks. Although all of them was shown to possess the full complement of neural stem cells (NSCs) characteristics [22, 32, 33, 61], we show how each GSCs line was endowed with distinct ability to perpetuate and expand across glioblastoma sub-classes. Integrating and confirming their molecular features, we retrieved that GSCs derived from the classical subtype displayed a higher clonal efficiency and were fast growing *ex-vivo* and much less invasive (Fig. 3B-D). Instead, mesenchymal and proneural GSCs inherently presented the lowest expansion rate and self-renewal capacity, while invading much more efficiently. Focusing on the in vivo functional analyses, despite being all of GSCs lines tested able to produce tumors that closely resemble the main architectural features of glioblastoma [22, 32, 33, 35], we found that these cells differed quite significantly across subtype in their inherent ability to spread throughout the brain parenchyma. The xenografting of mesenchymal GSCs established intracranial human GBMs with a strikingly faster and broader infiltration pattern than those of classical GSCs, which gave rise to larger, more defined neoplastic masses in the mouse brain (Fig. 4C). The proneural GSCs seemed to display a pattern similar to that of mesenchymal GSCs. This not only provided us a better understanding of the intrinsic heterogeneity of GSCs’ basic physiology but also the evidence that GBM subgroups contain GSCs inherently endowed with differential self-renewal ability, thus underpinning their functional inter-cluster variances. Furthermore, the lower self-renewal capacity and expansion rate and the higher invasion efficiency retrieved in GSCs from mesenchymal and proneural subtype, as compared to that one of classical cluster, might also explain the variation in response to therapy of their patients of origin.

The search for innovative, more effective therapies for GBM is now focusing on targeting the relatively rare GSCs cells, that are responsible for tumor growth, resistance to treatments and recurrence. From this perspective, having observed the existence of distinct GSCs’ stemness-related phenotypes across GBM cluster, on their primary specimen and clinical data of the donor patient, we also found that the inherently, stemness-related, different abundance of *DCX* and *EGFR* perfectly discriminated GBM patients predicting their stratification into TCGA clusters and their survival (Fig. 5), thus identifying potential subtype-associated biomarkers for patient-tailored diagnostic and prognostic purposes.

In considering the heterogeneous sensitivity of GBM patients at large to standard treatments, which may mostly come from belonging to functionally and molecularly distinct clusters, this also allow to recognize those tumors that may be the most appropriate target for different therapeutic protocols, opening new and exciting possibilities for the definition of novel patient-specific drug-responsiveness protocols that may help to devise appropriate patient-selective therapies, such as patient-tailored chemotherapy regimen, differentiation therapies and combined protocols.

## Conclusion

In this study, we identified distinct GSCs cells subsets within GBM patients’ clusters, which are inherently endowed with different self-renewal, invasive and tumorigenic ability underpinning inter-cluster variance together with diverse clinical outcome and response to therapy. Two biomarkers emerged selectively enriched among these different states of stemness, DCX and EGFR, are capable of predicting GBM patients’ clusters, their overall survival and drug sensitivity. These findings may not only promote our understanding of the intrinsic intra-heterogeneity of GBM cases and their GSCs but also provide new potential critical targets opening new and exciting possibilities for GBM prognosis and more effective, individual patient-specific treatment.

### Supplementary Information

**Additional file 1.**

**Additional file 2.**

**Additional file 3.**

**Additional file 4.**

**Additional file 5.**

**Additional file 6.**

**Additional file 7.**

## Data Availability

Transcriptome, Cytoscan array and Targeted sequencing raw data are available in the Arrayexpress repository under the accession codes E-MTAB-12962, E-MTAB-12963 and E-MTAB-12964, respectively.

## Abbreviations

GBM: Glioblastoma
GSCs: Glioblastoma stem cells
IDH1: Isocitrate dehydrogenase 1
TCGA-CL: Classical Cancer Genome Atlas
TCGA-PN: Proneural Cancer Genome Atlas
TCGA-MS: Mesenchymal Cancer Genome Atlas
NSCs: Neural stem cells
CNV: Copy number variation
SNVs: Single nucleotide variants
FDR: False discovery rate
TERT: Telomerase Reverse Transcriptase
EGFR: Epidermal Growth Factor Receptor
DCX: Doublecortin
DPT: Days post transplantation
