# Supplementary material for: Different states of stemness of glioblastoma stem cells sustain glioblastoma subtypes indicating novel clinical biomarkers and high-efficacy customized therapies
Source: J Exp Clin Cancer Res. 2023 Sep 21;42:244. doi: 10.1186/s13046-023-02811-0 (PMC10512479; doi:10.1186/s13046-023-02811-0)
Supplement: Supplementary file 1 — Additional file 1. [file 13046_2023_2811_MOESM1_ESM.pdf]

Different state of stemness of glioblastoma stem cells sustain human glioblastoma subtypes indicating novel clinical biomarkers and high-efficacy customized therapies

Alberto Visioli, Nadia Trivieri, Gandino Mencarelli, Fabrizio Giani, Massimiliano Copetti, Orazio Palumbo, Riccardo Pracella, Maria Grazia Cariglia, Chiara Barile, Luigi Mischitelli, Amata Amy Soriano, Pietro Palumbo, Federico Legnani, Francesco DiMeco, Leonardo Gorgoglione, Graziano Pesole, Angelo L. Vescovi and Elena Binda.

Supplementary information

Supplementary Methods

Supplementary Figure 1-4

Supplementary Table 1-6

Supplementary Methods

Invasion assays

Invasion assays were performed in 6-well Transwell chambers (Corning) (1). The upper side of the filters was washed with Acetic Acid and then coated with Cultrex (Trevigen). 2,5 x 10^5 cells were seeded onto the layer coated with Cultrex in the NeuroCult NS-A medium (Stem Cell Technologies). Ten to twelve days after plating, cells were fixed and stained using Hemacolor Rapid Staining Kit (Merck-Millipore). Cells on the upper side of the filters were mechanically removed, and those migrated onto the lower side were counted by microscope (2- 4).

Clonogenic Assay

24 wells (Corning) were coated with 200 ul of Poly-D-Lysin (Sigma-Aldrich) for 24 hours. 1x10^3 GSCs cells derived from the dissociation of neurospheres were then placed in each coated well in Neurocult NS-A medium (Stem Cell Technologies). After 7 to 10 days, the number of secondary spheres formed was counted by microscope (2- 5).

Nucleic acid purification and quantification

Genomic DNA was extracted using Blood and Cell Culture DNA Midi Kit (Qiagen) following manufacturer's protocol. To evaluate the quality and purity of the extracted DNA we used both the absorbance A260/280 and gel electrophoresis. Total RNA from fresh post-surgery tissues and GSCs cells was extracted using RNeasy Mini kit (Zymo Research) and quantified by the NanoDrop ND-2000 (Thermo Scientific) and the RNA integrity was assessed using Agilent Bioanalyzer 2100 (Agilent Technologies) (2-5).

Targeted sequencing and mutation calling analysis

To get insight into the genomic events differentiating the subtypes, 31 high-grade glioma tissues, matched blood DNA (available from 27 patients) and their 32 derivative GSCs lines

were sequenced by Illumina MiSeq Platform (Illumina) (3-5). Protein-coding regions of the following 27 genes typically altered in GBM and/or key regulators in stemness and differentiation were examined (6-8): *ATRX, KRAS, EPHA2, NRAS, IDH1, CTNNB1, PIK3CA, WNT5A, PDGFRA, PIK3R1, TERT, BRAF, EGFR, MET, CDKN2A, BMP4, BMPR1A, BMPR1B, BMPR2, MGMT, PTEN, CCND2, CDK4, RB1, ERBB2, NF1, TP53*. Illumina Design Studio was employed to design a Truseq Custom Amplicon Kit and a AmpliSeq kit (Illumina) and genomic DNA from GBM primary tissues, their matched peripheral blood and derivative GSCs lines was used to prepare libraries sequenced by Illumina MiSeq platform. After sequencing, the raw data were processed using the MiSeq Reporter software (Illumina Inc) to be demultiplexed and the resulting sequences were written in FASTQ format. GATK and VarScan were used to identify germline and somatic single nucleotide variants (SNVs) and small insertions and deletions (indels) in GBM patient's tissues with their matched blood as in (4, 9, 10). For the samples lacking matched blood DNA, in order to exclude germline variants, nucleotide variants were identified using GATK HaplotypeCaller, which was calibrated with a set of normal samples as a surrogate for the missing bloods. AnnoVar algorithm (11) was utilized to annotate somatic variants, which combined information from several genomic and protein databases (GENECODE, UniProt, dbNSFP) along with variant databases relevant to cancer (COSMIC, ClinVar) and non-cancer (dbSNP, 1000 Genomes, Kaviar, Haplotype Reference Consortium, Exome Aggregation Consortium, NHLBI Exome Variant Server). The variants with direct impacts on the protein sequence (missense, truncating, stoploss, splicing variants, frameshift, and in-frame indels) were selected. Variants present in non-cancer databases with a minor allele frequency ≥ 0.05 were categorized as germline polymorphisms and subsequently removed. Multiple prediction algorithms (MutationTaster2, Polyphen2, Provean, and SIFT, FATHMM-Indel, Provean, SIFT-Indel, and VEST-Indel) were utilized to determine the functional effect of missense SNVs and in-frame indels and their pathogenicity

(12-15). All somatic variants classified as pathogenic mutations were finally validated by Sanger sequencing using the ABI Prism BigDye Terminator v3.1 Cycle Sequencing Kit (Applied Biosystems) and the results analyzed as previously described in (4, 5). All primer used are listed in the Supplementary Table 2.

RT-qPCR. cDNA was obtained from total RNA and qPCR performed as described in (3-5). Delta Ct method was used to determine the gene expression profile by quantifying mRNA levels relative to a control sample. The relative expression level is depicted in box and whiskers plot that displays the median (line inside the box), first quartile (Q1) and third quartile (Q3). The whiskers represent the minimum and maximum value.

DNA copy number analysis

Somatic CNVs of all samples was examined by SNP array by the CytoScanHD array platform (Affymetrix) according to manufacturer's protocol and data analyzed with Partek Genomics Suite 7.0 as previously described (3, 16). Genomic regions recurrently amplified or deleted regions were recognized and associated to each patient across subtypes with GISTIC2.0 (17). Data were graphically depicted as circos plots.

Microarray analysis of gene expression

Gene expression profiling of GBM tissues (n=58) and of GSCs lines (n=27) was performed with total RNA by the Affymetrix GeneChip® Human Transcriptome Array 2.0 (Affymetrix) according to the manufacturer's instructions. Quality control steps and analysis of .CEL files was performed using R (ver. 3.6.0) and differentially expressed genes were then examined with Partek Genomics Suite package ver. 7.0 (3-5, 18). The Benjamini–Hochberg false discovery rate (FDR) was employed to correct the P-values (q-values). Only genes with a q-value<0.05

were considered differentially enriched. The most significantly enriched biological functions and diseases were identified by Ingenuity Pathway Analysis (IPA; Qiagen, http://www.ingenuity.com/) and R software (3-5) and graphed by bubble plot.

***IDH1*, *EGFRvIII* and *TERTp* mutational status**. The most common mutations in *IDH1* and the promoter of *TERT* gene were determined in all samples using condition as in (4, 19). For analysis of *EGFRvIII* status, complementary DNA (cDNA) was synthesized used a SuperScript™ III Reverse Transcriptase (Thermo Fisher) following the manufactory protocol, and PCR was performed as described in (20). The PCR products for *EGFR* (1044bp) and *EGFRvIII* (243bp) were visualized using QIAxcel (an automated capillary electrophoresis system by Qiagen). Long-range PCR using 200 ng of genomic DNA was performed as describe in (21) and PCR products were separated and visualized by 0.8% agarose gel electrophoresis.

Immunohistochemistry and immunofluorescence

Immunohistochemistry and immunofluorescence analysis of GBM tissues and GSCs cells were performed as previously described (2-5, 18). The following primary antibodies were used: mouse anti-human Nuclei (1:100; Merck-Millipore), rabbit anti-GFAP (1:200; Dako), mouse anti-Galactocerebroside C (1:200; Merck-Millipore), mouse anti-Tubulin beta III (1:400; Biolegend), rabbit anti-Melk (1:100; Sigma-Aldrich), mouse anti-SSEA1 (1:50; BD Bioscience), mouse anti-CD44 (1:50; BD Pharmigen). As secondary antibodies, goat anti-mouse AlexaFluor488 (1:1000; ThermoFisher Scientific), goat anti-mouse AlexaFluor546 (1:1000; ThermoFisher Scientific), donkey anti-goat AlexaFluor488 (1:1000; ThermoFisher Scientific), goat anti-rabbit AlexaFluor546 (1:1000; ThermoFisher Scientific).

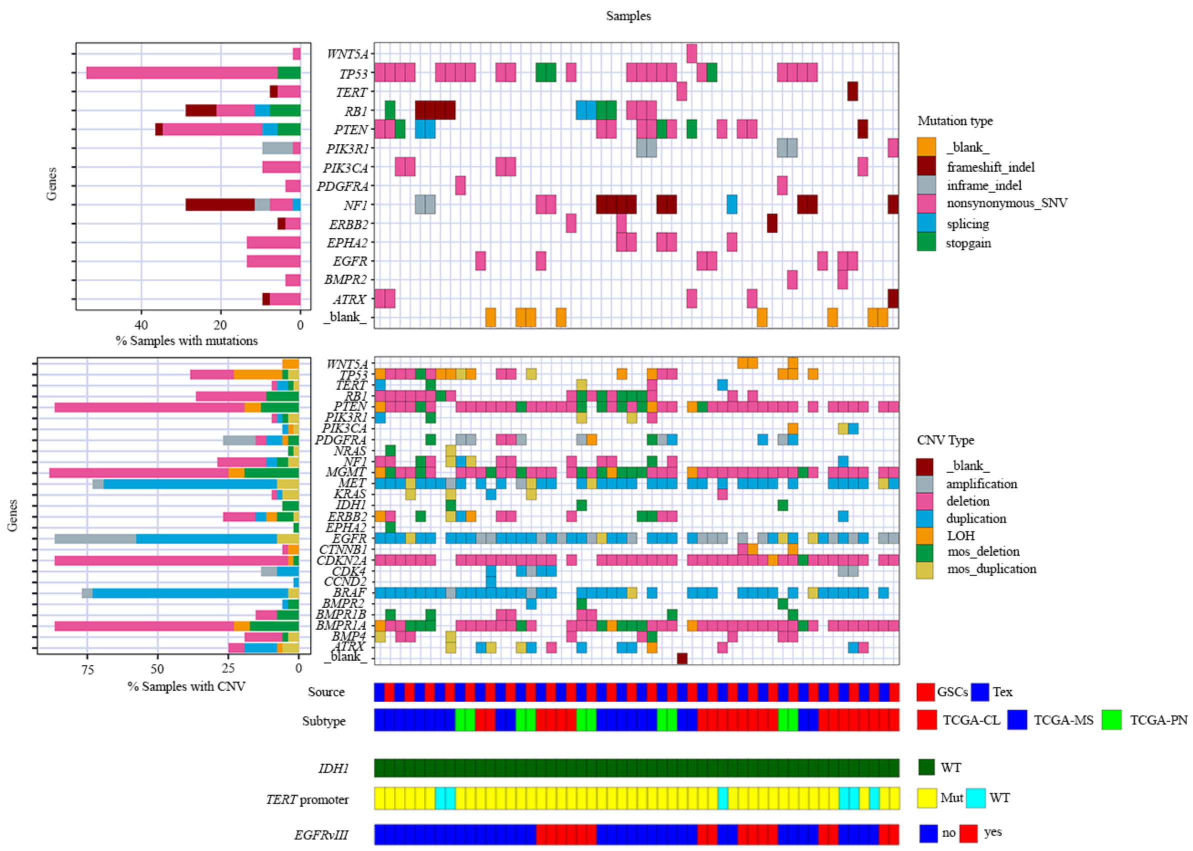

Supplementary Figure 1. Landscape of genetic alterations in GBM patients' tissue and GSCs line pairs. Integrated matrix of 26 GBM tissues and GSCs lines pairs showing gene variants and significant CNVs and allelic frequencies (loss of heterozygosity; LOH, amplification, deletion, duplication, mosaicisms). Columns report number and type of the integrated genomic profile for each sample.

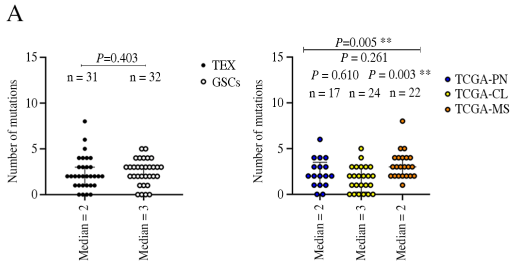

B

Gene	TCGA-CL (n=24)	TCGA-MS (n=22)	TCGA-PN (n=17)	Total No. of Mutated Samples
**EGFRvIII**	**14 (58%)** ***	**0 (0%)*****	3 (18%)	17
**MET**	0 (0%)	0 (0%)	1 (6%)	1
**BMPR2**	1 (4%)	0 (0%)	1 (6%)	2
**ATRX**	2 (8%)	3 (14%)	2 (12%)	7
**TERT**	1 (4%)	1 (5%)	0 (0%)	2
**ERBB2**	2 (8%)	2 (9%)	0 (0%)	4
**EPHA2**	1 (4%)	4 (18%)	5 (29%)	10
**PDGFRA**	0 (0%)	0 (0%)	**3 (18%)** *	3
**PIK3CA**	1 (4%)	**6 (27%)** **	0 (0%)	7
**RB1**	**1 (4%)** **	**12 (55%)** ***	3 (18%)	16
**PIK3R1**	1 (4%)	3 (14%)	2 (12%)	6
**EGFR**	**8 (33%)** ***	**0 (0%)** *	0 (0%)	8
**NF1**	5 (21%)	9 (41%)	3 (18%)	17
**PTEN**	5 (21%)	10 (45%)	5 (29%)	20
**TP53**	7 (29%)	13 (59%)	8 (47%)	28

(* P<0.05, ** P<0.01, *** P<0.001, two-sided Fisher's exact test)

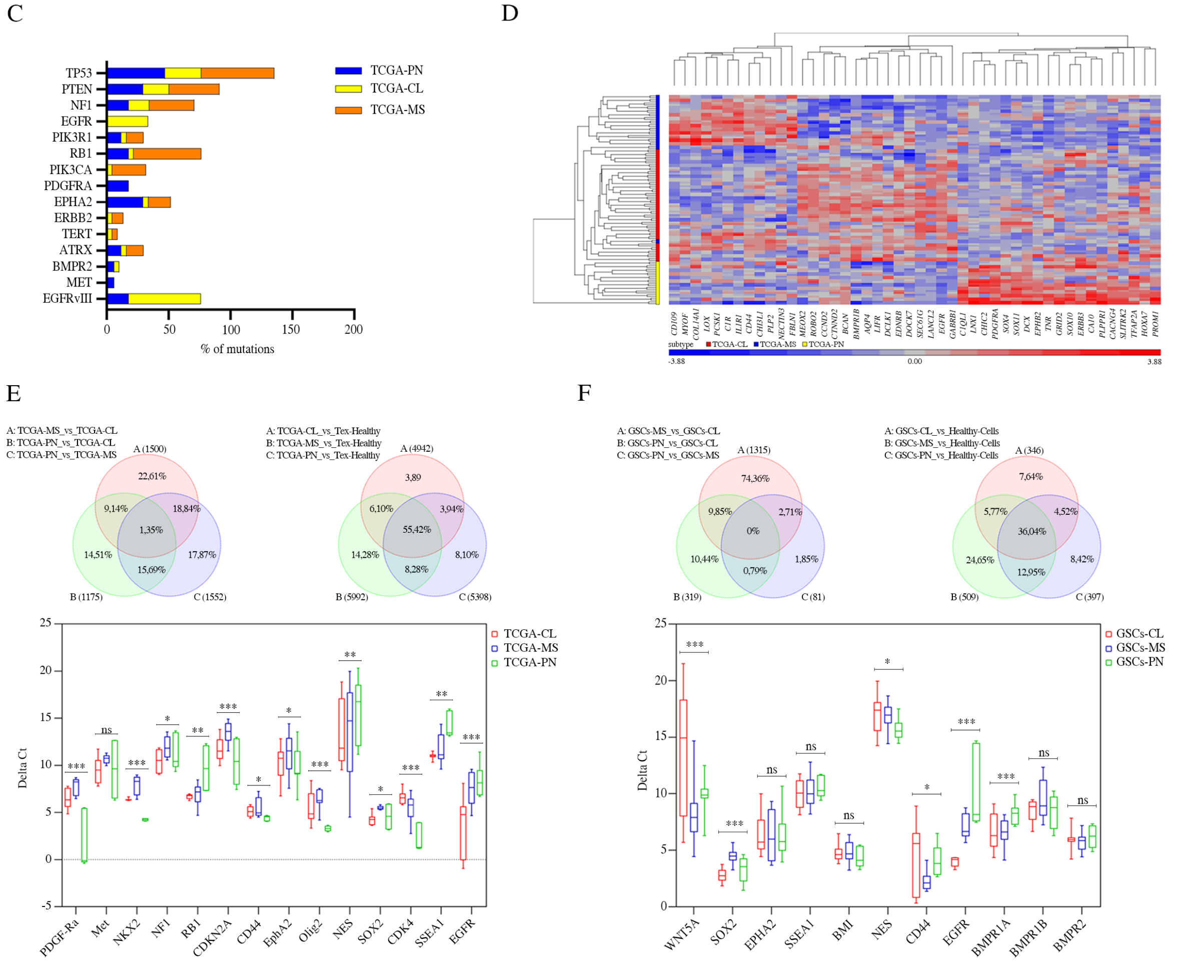

Supplementary Figure 2. Overview of the identification of subtype-specific molecular profiles for GBM patients and their derivative GSCs. A. Scatter plot showing the comprehensive number of somatic mutations (SNVs and indels) occurring in high-grade patients (TEX; n=31) and their sibling GSCs (GSCs, n=32) P=0.403, two-sided Mann-Whitney-Wilcoxon test; MWW (left) and across subtypes (right) (n=17 TCGA-PN, n=24 TCGA-CL and n=22 TCGA-MS samples) P=0.005, Kruskal-Wallis test. **B-C.** Distribution of the frequently mutated genes, in terms of total number of mutated samples (**A**) and percent of

mutations (**B**), in GBM tissues and their derivative GSCs lines across subtypes. ***$P<0.001$, **$P<0.01$, *$P<0.05$, two-sided Fisher's exact test. **D.** Unsupervised hierarchical clustering analysis based on selected genes differentially expressed when comparing GBM patients across subtypes ($n=30$ TCGA-CL, $n=16$ TCGA-MS and $n=12$ TCGA-PN), which clearly identifies each molecular cluster in an unsupervised manner. Samples are coded by color. A dual-color code represents genes over- (red) and under-represented (blue), respectively. **E.** Venn diagrams confirming the distinctive gene expression profile of each molecular subtype (top, left). Overlaps of differentially expressed genes were shown to be increased when comparing GBM tissues to normal brain control (top, right). *q*value<0.05 and fold change=2. Bottom: Quantification of mRNA level for the indicated genes in GBM patients' post-surgery specimens across subtypes by qPCR. Data are graphically depicted by boxplot, which shows median ± IQR. ***$P<0.001$, **$P<0.01$, *$P<0.05$, ns not significant, Kruskal-Wallis test. **F.** Venn diagram reporting the peculiar transcriptional profile of GSCs lines across GBM subtypes (top, left) and the common genes associated to GSCs as compared to human normal neural stem cells (NSCs) (top, right). Bottom: mRNA expression level for the indicated markers in GSCs across subtypes, as quantified by qPCR. Data are graphically depicted by boxplot. ***$P<0.001$, *$P<0.05$, ns not significant, Kruskal-Wallis test.

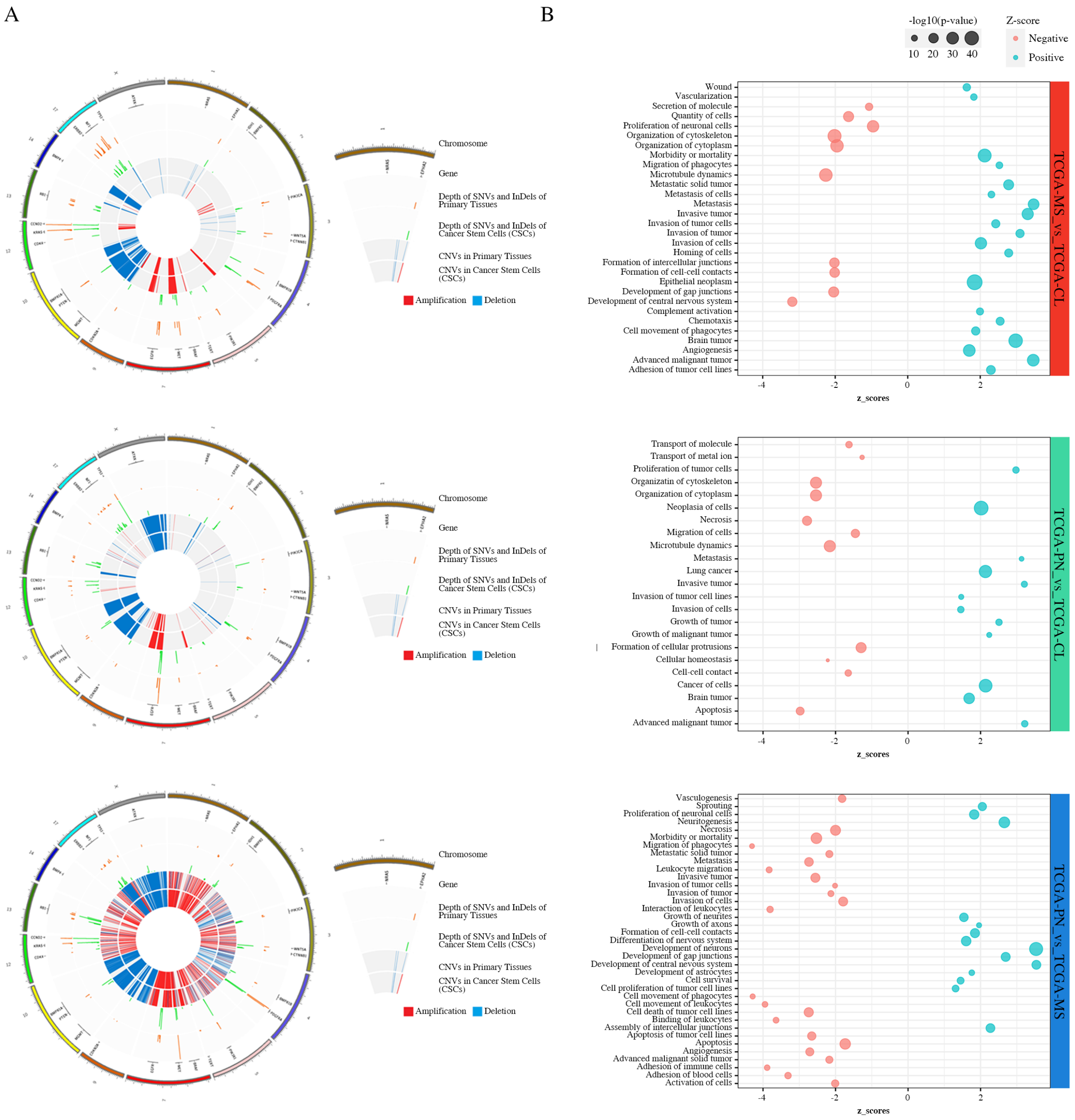

Supplementary Figure 3. Identification of a mutual aberrated profile in GBM tissues and their sibling GSCs cells. A. Circos plot for GBM patients' tissues from mesenchymal (top), classical (middle) and proneural (bottom) subtypes versus their derivative GSCs lines revealing similar genetic abnormalities, distinctive for each subcluster. In each case, the outer track provides mutations inside the circle and the inner track shows CNVs. Blue and red represent deletions and amplifications, respectively. **B.** Bubble plots revealing a subtype-specific diseases and functions signature for GBM patients similar to that one reported in Figure 2 for

their derivative GSCs. The enriched transcripts and their biological functions from the comparison of TCGA-MS and TCGA-PN patients vs. TCGA-CL ones are shown to be mainly involved in *invasion*, *metastasis* and *angiogenesis*, while the most downregulated cellular functions are related to *proliferation*, *organization of the cytoskeleton* and *apoptosis*. By comparing TCGA-PN vs. TCGA-MS cases, the over-represented processes are related to *neurogenesis*. Circles sizes representing $-\log_{10}(P\text{-value})$ and color change, red or blue, based on negative and positive z-score respectively.

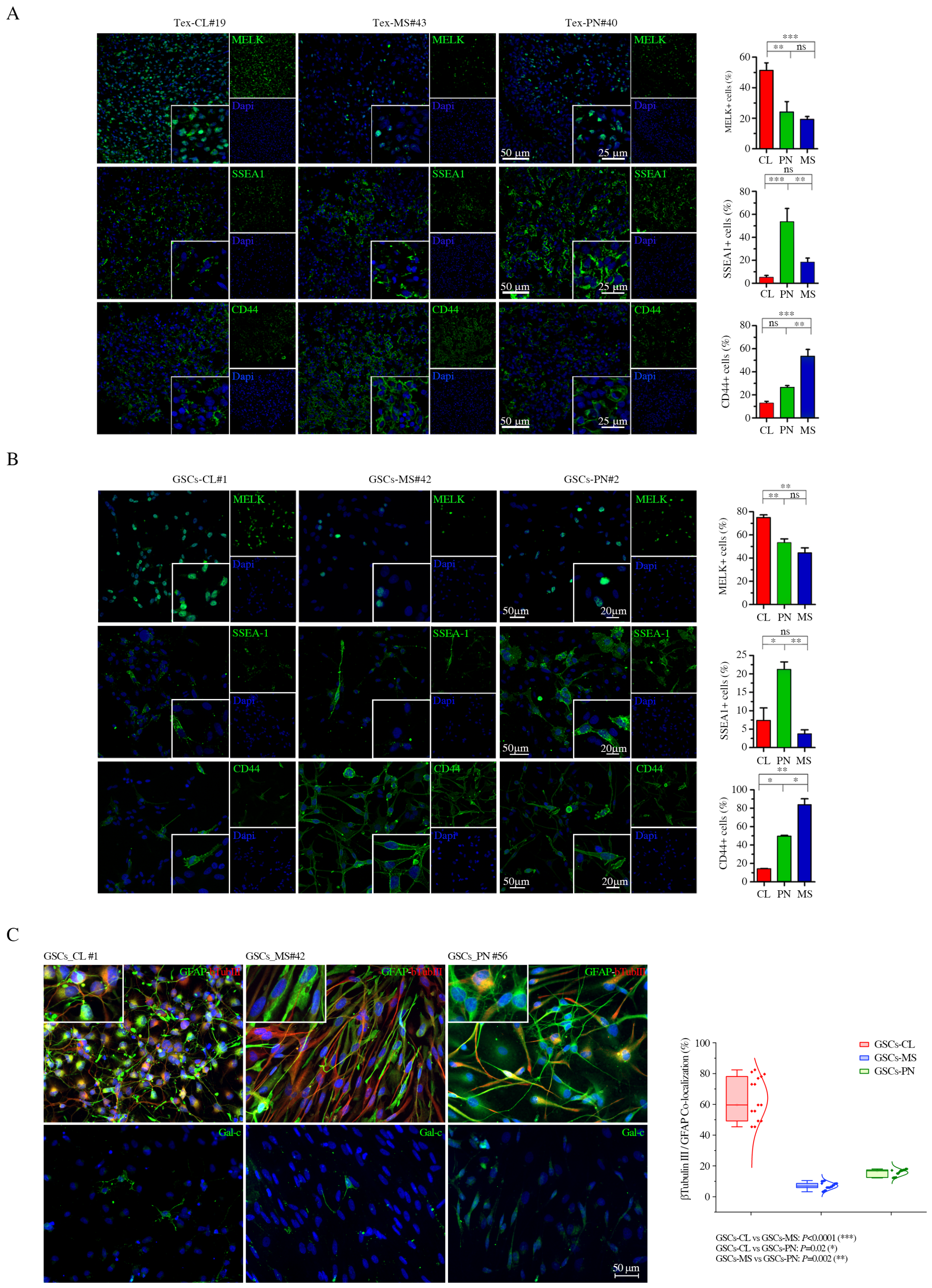

Supplementary Figure 4. Immunofluorescence validation of the subtypes-specific signature. A-B. Representative confocal immunolabeling of surgery specimens (**A**) and their

derivative GSCs cells (**B**) reporting strong MELK immunoreactivity (green) in numerous cells within GBM and GSCs from TCGA-CL subtype with respect to a fewer immunoreactive cells in TCGA-MS and TCGA-PN tissues and cells. Widespread positivity for the markers of stemness SSEA1(green) was observed in proneural samples, whereas CD44 (green) displayed much more intense immunoreactivity in mesenchymal tissues and GSCs as compared to their classical and proneural counterpart. Insets: higher magnifications. Scale bars: 20μm, 25μm, 50μm. A quantitative analysis of the frequency of each marker is shown. Data are mean ± SEM. ****P*<0.0001, ***P*<0.01, **P*<0.05, ns not significant, one-way ANOVA test. **c.** Immunofluorescence representative images for glial (GFAP; green), neuronal (class III b-tubulin; red) and oligodendroglial (GalC; green) markers reporting GSCs' multipotential capacity across subtype. Several cells which promiscuously co-labeled with glial and neuronal markers are denoted (yellow) in TCGA-CL cultures as compared to TCGA-MS and TCGA-PN cells. Insets: higher magnification. *Bar*, 50um. Data are graphically depicted by boxplot. *P*-values from Kruskal-Wallis ANOVA dunn's test are shown.

References

1. Pennacchietti S, Michieli P, Galluzzo M, Mazzone M, Giordano S, Comoglio PM. Hypoxia promotes invasive growth by transcriptional activation of the met protooncogene. Cancer Cell. 2003 Apr;3(4):347-61. PubMed PMID: 12726861. eng.

2. Galli R, Binda E, Orfanelli U, Cipelletti B, Gritti A, De Vitis S, et al. Isolation and characterization of tumorigenic, stem-like neural precursors from human glioblastoma. Cancer Res. 2004 Oct;64(19):7011-21. PubMed PMID: 15466194. eng.

3. Binda E, Visioli A, Giani F, Trivieri N, Palumbo O, Restelli S, et al. Wnt5a Drives an Invasive Phenotype in Human Glioblastoma Stem-like Cells. Cancer Res. 2017 Feb;77(4):996-1007. PubMed PMID: 28011620. Epub 2016/12/23. eng.

4. Trivieri N, Visioli A, Mencarelli G, Cariglia MG, Marongiu L, Pracella R, et al. Growth factor independence underpins a paroxysmal, aggressive Wnt5a(High)/EphA2(Low) phenotype in glioblastoma stem cells, conducive to experimental combinatorial therapy. J Exp Clin Cancer Res. 2022 Apr 12;41(1):139. PubMed PMID: 35414102. PMCID: PMC9004109. Epub 20220412. eng.

5. Visioli A, Giani F, Trivieri N, Pracella R, Miccinilli E, Cariglia MG, et al. Stemness underpinning all steps of human colorectal cancer defines the core of effective therapeutic strategies. EBioMedicine. 2019 Jun;44:346-60. PubMed PMID: 31056474. Epub 2019/05/02. eng.

6. Wang J, Cazzato E, Ladewig E, Frattini V, Rosenbloom DI, Zairis S, et al. Clonal evolution of glioblastoma under therapy. Nat Genet. 2016 Jul;48(7):768-76. PubMed PMID: 27270107. PMCID: PMC5627776. Epub 20160606. eng.

7. Binda E, Reynolds BA, Vescovi AL. Glioma stem cells: turpis omen in nomen? (The evil in the name?). J Intern Med. 2014 Jul;276(1):25-40. PubMed PMID: 24708237. eng.

8. Verhaak RG, Hoadley KA, Purdom E, Wang V, Qi Y, Wilkerson MD, et al. Integrated genomic analysis identifies clinically relevant subtypes of glioblastoma characterized by abnormalities in PDGFRA, IDH1, EGFR, and NF1. Cancer Cell. 2010 Jan;17(1):98-110. PubMed PMID: 20129251. PMCID: PMC2818769. eng.

9. DePristo MA, Banks E, Poplin R, Garimella KV, Maguire JR, Hartl C, et al. A framework for variation discovery and genotyping using next-generation DNA sequencing data. Nat Genet. 2011 May;43(5):491-8. PubMed PMID: 21478889. PMCID: PMC3083463. Epub 2011/04/10. eng.

10. Koboldt DC, Zhang Q, Larson DE, Shen D, McLellan MD, Lin L, et al. VarScan 2: somatic mutation and copy number alteration discovery in cancer by exome sequencing. Genome Res. 2012 Mar;22(3):568-76. PubMed PMID: 22300766. PMCID: PMC3290792. Epub 2012/02/02. eng.

11. Wang K, Li M, Hakonarson H. ANNOVAR: functional annotation of genetic variants from high-throughput sequencing data. Nucleic Acids Res. 2010 Sep;38(16):e164. PubMed PMID: 20601685. PMCID: PMC2938201. Epub 2010/07/03. eng.

12. Choi Y, Chan AP. PROVEAN web server: a tool to predict the functional effect of amino acid substitutions and indels. Bioinformatics. 2015 Aug;31(16):2745-7. PubMed PMID: 25851949. PMCID: PMC4528627. Epub 2015/04/06. eng.

13. Ferlaino M, Rogers MF, Shihab HA, Mort M, Cooper DN, Gaunt TR, et al. An integrative approach to predicting the functional effects of small indels in non-coding regions of the human genome. BMC Bioinformatics. 2017 Oct;18(1):442. PubMed PMID: 28985712. PMCID: PMC5955213. Epub 2017/10/06. eng.

14. Hu J, Ng PC. SIFT Indel: predictions for the functional effects of amino acid insertions/deletions in proteins. PLoS One. 2013;8(10):e77940. PubMed PMID: 24194902. PMCID: PMC3806772. Epub 2013/10/23. eng.

15. Douville C, Masica DL, Stenson PD, Cooper DN, Gygax DM, Kim R, et al. Assessing the Pathogenicity of Insertion and Deletion Variants with the Variant Effect Scoring Tool (VEST-Indel). Hum Mutat. 2016 Jan;37(1):28-35. PubMed PMID: 26442818. PMCID: PMC5057310. Epub 2015/10/26. eng.

16. Schonberg DL, Lubelski D, Miller TE, Rich JN. Brain tumor stem cells: Molecular characteristics and their impact on therapy. Mol Aspects Med. 2014 Oct;39:82-101. PubMed PMID: 23831316. PMCID: PMC3866208. Epub 2013/07/04. eng.

17. Mermel CH, Schumacher SE, Hill B, Meyerson ML, Beroukhim R, Getz G. GISTIC2.0 facilitates sensitive and confident localization of the targets of focal somatic copy-number alteration in human cancers. Genome Biol. 2011;12(4):R41. PubMed PMID: 21527027. PMCID: PMC3218867. Epub 20110428. eng.

18. Trivieri N, Pracella R, Cariglia MG, Panebianco C, Parrella P, Visioli A, et al. BRAF V^{600E} mutation impinges on gut microbial markers defining novel biomarkers for serrated colorectal cancer effective therapies. J Exp Clin Cancer Res. 2020 Dec;39(1):285. PubMed PMID: 33317591. PMCID: PMC7737386. Epub 2020/12/14. eng.

19. Balss J, Meyer J, Mueller W, Korshunov A, Hartmann C, von Deimling A. Analysis of the IDH1 codon 132 mutation in brain tumors. Acta Neuropathol. 2008 Dec;116(6):597-602. PubMed PMID: 18985363. eng.

20. Mellinghoff IK, Wang MY, Vivanco I, Haas-Kogan DA, Zhu S, Dia EQ, et al. Molecular determinants of the response of glioblastomas to EGFR kinase inhibitors. N Engl J Med. 2005 Nov;353(19):2012-24. PubMed PMID: 16282176. eng.

21. Frederick L, Eley G, Wang XY, James CD. Analysis of genomic rearrangements associated with EGFRvIII expression suggests involvement of Alu repeat elements. Neuro Oncol. 2000 07;2(3):159-63. PubMed PMID: 11302336. PMCID: PMC1920493. eng.
